# Spontaneous inferior epigastric artery hemorrhage in a COVID-19 patient with membranous nephropathy on anticoagulant therapy: a Case Report

**DOI:** 10.3389/fmed.2025.1728723

**Published:** 2026-01-02

**Authors:** Nianzong Hou, Guoxiang Xu, Lin Wang, Yun Zhang, Yong Yu, Lin Zhu, Weiwei Song, Rumin Zhang, Kai Wang

**Affiliations:** 1 Center of Translational Medicine, Zibo Central Hospital, Binzhou Medical University, Zibo, Shandong, China; 2 Center of Gallbladder Disease, Shanghai East Hospital, Institute of Gallstone Disease, School of Medicine, Tongji University, Shanghai, China; 3 Department of Critical Care Medicine, Zibo Central Hospital, Binzhou Medical University, Zibo, Shandong, China; 4 Department of Gastrointestinal Surgery, Zibo Central Hospital, Binzhou Medical University, Zibo, Shandong, China

**Keywords:** anticoagulant therapy, COVID-19, inferior epigastric artery, membranous nephropathy, spontaneous hemorrhage

## Abstract

**Background:**

The management of Coronavirus disease 2019 (COVID-19) is complicated by coagulopathies that increase both thrombotic and hemorrhagic risks, particularly in patients with comorbidities such as membranous nephropathy (MN) who require anticoagulation. Spontaneous inferior epigastric artery (IEA) hemorrhage is a rare but life-threatening complication in this setting.

**Case presentation:**

A 63-years-old woman with M-type phospholipase A2 receptor (PLA2R)-positive MN was admitted for COVID-19 pneumonia and acute respiratory distress syndrome (ARDS). Despite prophylactic anticoagulation with dalteparin, her respiratory status deteriorated, requiring intensive care. On day 24, she developed sudden hemorrhagic shock due to a rectus sheath hematoma from spontaneous IEA rupture, confirmed by imaging and surgical exploration. The hemorrhage was managed with ligation, transfusion, and discontinuation of anticoagulation. Her recovery was marked by resolution of pulmonary and hemorrhagic complications by day 46, and 3-months follow-up showed no recurrence of bleeding or thrombotic events.

**Conclusion:**

This case highlights the critical balance between thromboprophylaxis and bleeding risk in COVID-19 patients with MN. It underscores the need for individualized anticoagulation strategies, pharmacodynamic monitoring, and multidisciplinary decision-making to mitigate risks in this high-risk population. The interplay of COVID-19-induced coagulopathy, renal impairment, and immunosuppressive therapy amplifies both thrombotic and hemorrhagic tendencies, necessitating extreme vigilance in clinical management.

## Introduction

The novel coronavirus disease 2019 (COVID-19), caused by severe acute respiratory syndrome coronavirus 2 (SARS-CoV-2), has been associated with a wide spectrum of clinical manifestations, ranging from mild respiratory symptoms to severe acute respiratory distress syndrome (ARDS) and multi-organ dysfunction (1). While pulmonary complications dominate the clinical picture, extrapulmonary involvement–including renal, cardiovascular, and hematologic systems–has been increasingly recognized (2). Elderly patients and those with comorbidities such as chronic kidney disease, cardiovascular disorders, or immunosuppression are particularly vulnerable to severe outcomes (3). A hallmark of severe COVID-19 is the dysregulation of coagulation and fibrinolysis, often leading to a hypercoagulable state characterized by microthrombosis, pulmonary embolism, and ischemic organ damage. Consequently, anticoagulant therapy has become a cornerstone of management in hospitalized patients to reduce thrombotic complications and mortality. However, the use of anticoagulants, especially at therapeutic doses, is not without risks. Bleeding complications, though less common, can be life-threatening and pose significant clinical challenges (4, 5).

Spontaneous hemorrhage of the inferior epigastric artery (IEA) is a rare but potentially catastrophic event. It most frequently occurs iatrogenically due to abdominal procedures such as laparoscopy, percutaneous drainage, or subcutaneous injections. Spontaneous cases are exceedingly rare and often associated with underlying coagulopathies, renal insufficiency, or vascular fragility. In the context of COVID-19, where both the disease itself and anticoagulant therapy can alter coagulation dynamics, the risk of spontaneous bleeding may be heightened (6–8). The pathophysiological interplay between SARS-CoV-2 infection and hemostatic disturbances involves endothelial injury, inflammatory cytokine release, and dysregulation of both procoagulant and anticoagulant pathways, creating a fragile balance that may tip toward hemorrhage in certain clinical scenarios.

This risk is particularly pronounced in patients with pre-existing renal conditions such as membranous nephropathy (MN). MN not only predisposes to a hypercoagulable state through urinary loss of anticoagulant proteins but also impairs drug metabolism and excretion, potentially leading to unexpected accumulation of anticoagulants even at prophylactic doses. Furthermore, the immunosuppressive regimens often required for MN management, particularly corticosteroids, can exacerbate vascular fragility and impair platelet function, creating a perfect storm for hemorrhagic complications.

Here, we present a case of spontaneous IEA rupture leading to a rectus sheath hematoma (RSH) in a 63-years-old woman with COVID-19 pneumonia, ARDS, and membranous nephropathy who was receiving prophylactic anticoagulation. This case highlights the delicate balance between thromboprophylaxis and hemorrhage risk in COVID-19 patients with renal comorbidities, and underscores the need for vigilant monitoring and individualized management. It also illustrates the critical importance of considering patient-specific factors such as renal function, comorbid conditions, and concomitant medications when formulating anticoagulation strategies in complex COVID-19 cases.

## Case presentation

**FIGURE 1:**
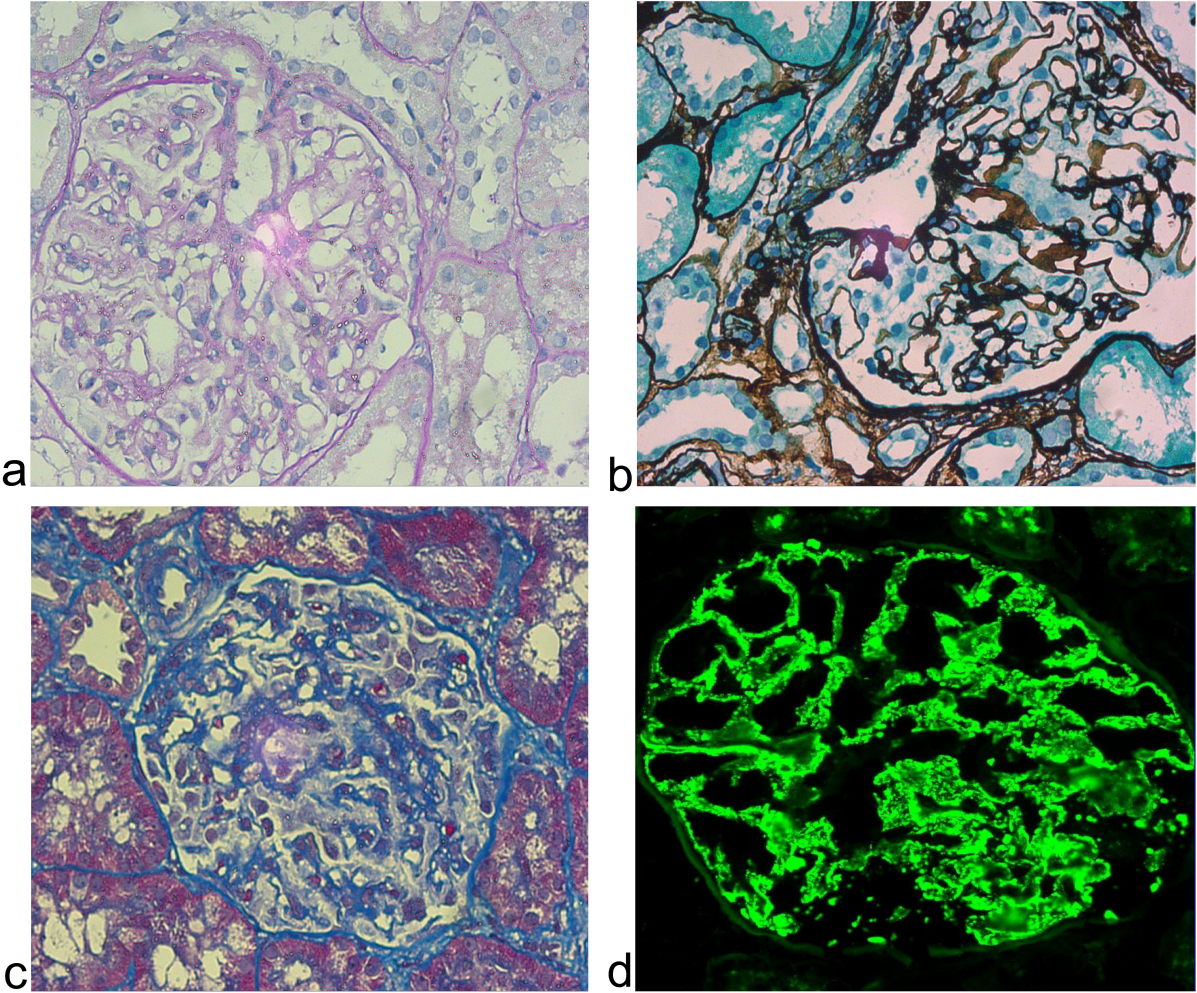
Pathological features of membranous nephropathy in a renal biopsy specimen of the patient (magnification ×400). **(a)** HE staining reveals a glomerulus with mild mesangial hypercellularity, capillary wall thickening, and segmental vacuolar degeneration, accompanied by a lightly stained mesangial matrix. **(b)** PASM staining demonstrates irregularly thickened basement membranes exhibiting alternating dark-brown (“spike”) and pale-stained areas, characteristic of subepithelial immune deposits. **(c)** PLA2R immunohistochemistry shows diffuse granular brown deposits along capillary walls, confirming PLA2R-associated membranous nephropathy. **(d)** IgG immunofluorescence displays intense green granular positivity along capillary loops, consistent with IgG deposition. PASM, Periodic Acid-Silver Methenamine; PLA2R, M-type phospholipase A2 receptor.

**FIGURE 2:**
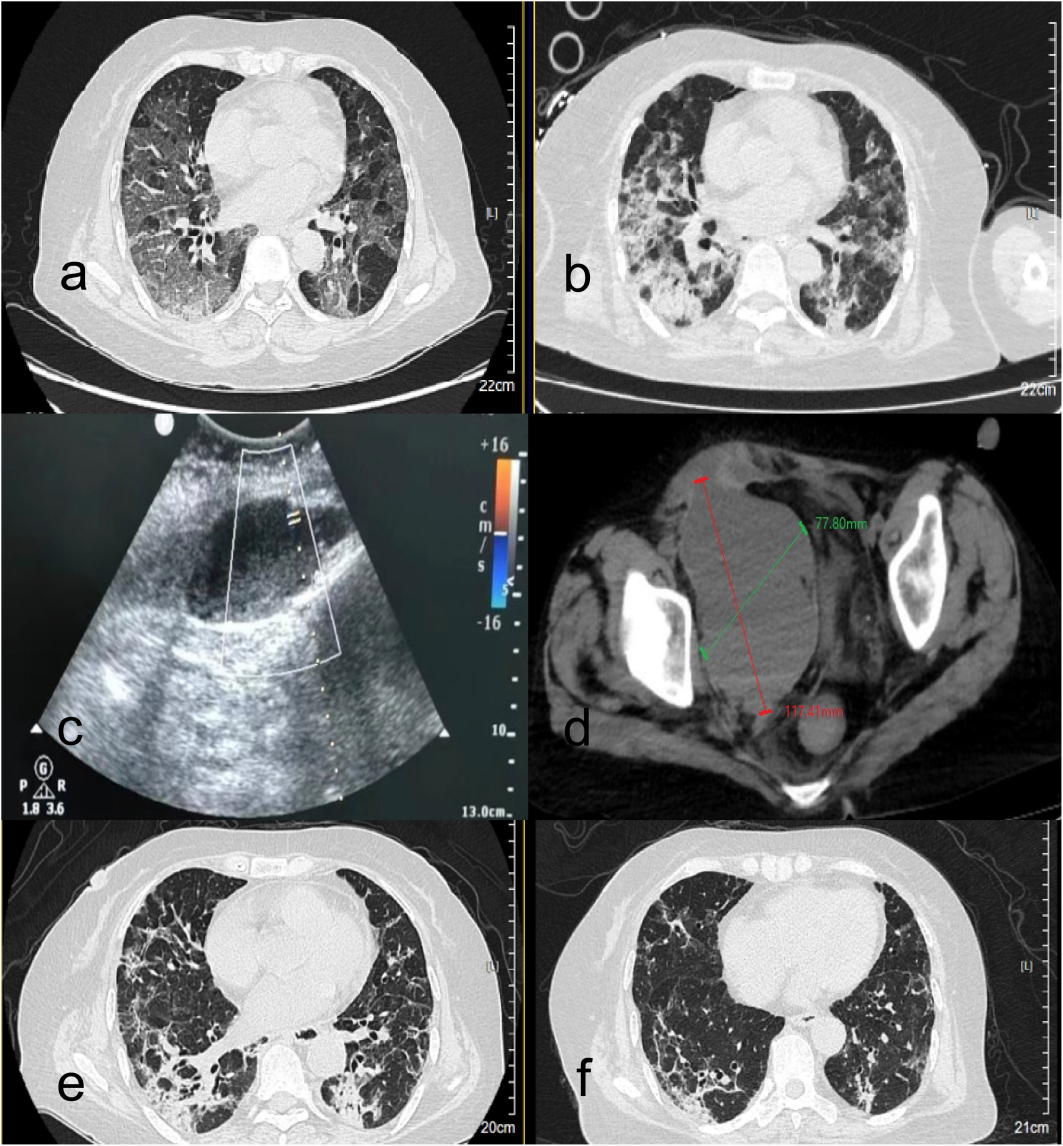
Radiological findings in a COVID-19 patient with membranous nephropathy during hospitalization. **(a,b)** Serial axial chest CT images (lung window). **(a)** Non-contrast CT on admission (day 1) reveals extensive bilateral multifocal ground-glass opacities and consolidations, predominantly distributed in peripheral and subpleural regions. **(b)** Follow-up CT on day 22 after treatment shows marked resolution of pulmonary opacities. **(c,d)** Imaging of rectus sheath hematoma. **(c)** Bedside ultrasonography demonstrates a cystic-solid mass extending from the rectus abdominis into the pelvis, with a maximum depth of 113 mm and poor sound transmission. Color Doppler imaging shows blood flow signals along the posterior wall of the rectus abdominis anterior to the cystic mass; pulse-wave Doppler reveals an arterial spectrum with a peak velocity of 30 cm/s. **(d)** Abdominal CT depicts a heterogeneous-density hematoma adjacent to the right rectus abdominis, extending into the pelvic cavity, measuring 117.41 mm in longitudinal and 77.80 mm in transverse diameters. **(e,f)** Follow-up axial chest CT (lung window) on day 35 **(e)** and day 46 **(f)**, demonstrating continued resolution of pulmonary opacities, with minimal residual subpleural ground-glass infiltrates and near-complete absorption of consolidations.

A 63-years-old Han Chinese woman was admitted to the respiratory department of Zibo Central Hospital on June 24, 2023, presenting with an 8-days history of fever and a 5-days history of progressive chest tightness. Her medical history included M-type phospholipase A2 receptor (PLA2R)-positive membranous nephropathy (MN), confirmed by renal biopsy in January 2022 (Figure 1), for which she was maintained on prednisone 10 mg daily. She also had a history of hypertension, specifically classified as grade 1 hypertension. The patient had no history of communicable or mental illnesses, and no significant family history of inherited diseases was reported. On admission, she was alert but in mild respiratory distress. Lung auscultation revealed coarse breath sounds with bilateral basilar crackles. Abdominal examination was unremarkable. SARS-CoV-2 PCR was positive. Chest computed tomography (CT) showed bilateral infiltrates consistent with COVID-19 pneumonia (Figure 2a). Laboratory findings revealed elevated inflammatory markers, along with impaired renal function evidenced by increased urea nitrogen at 8.24 mmol/L (normal range: 2.6–7.5 mmol/L) and creatinine at 109.0 μmol/L (normal range: 45–84 μmol/L). The patient also presented with hypokalemia, as indicated by a serum potassium level of 3.06 mmol/L (normal range: 3.5–5.3 mmol/L), and significant proteinuria, with a 24-h urinary protein measurement of 1.41 g (normal range: 0–0.15 g/24 h). Coagulation profile and other routine tests were within normal limits. The patient received antiviral therapy (Nirmatrelvir/Ritonavir), prophylactic anticoagulation with dalteparin (3000 IU q12 h), antibiotics, and high-flow nasal cannula oxygen.

On hospital day 8, she developed progressive hypoxemia requiring transfer to theintensive care unit (ICU), intubation, and mechanical ventilation. Her anticoagulation was still dalteparin 3000 IU q12 h based on critical illness and high thrombotic risk. Bronchoscopic sampling was performed to obtain bronchoalveolar lavage (BAL) fluid, which was subsequently analyzed by targeted next-generation sequencing (tNGS) for pathogen identification. The tNGS method employed an ultra-multiplex Polymerase Chain Reaction amplification system designed to enrich specific genomic sequences of clinically relevant pathogens. Following library preparation, high-throughput sequencing was conducted, and the resulting data were analyzed using a bioinformatic pipeline aligned to a curated microbial genomic database. The tNGS panel covered a predefined spectrum of 288 pathogens, including bacteria, fungi, viruses, and parasites, providing a comprehensive and targeted approach for detecting common and opportunistic respiratory pathogens. Based on high-confidence identification of genomic sequences, *Aspergillus fumigatus* and Herpes Simplex Virus (HSV) were detected, prompting targeted antifungal (voriconazole) and antiviral (acyclovir) therapy. We discontinued Prednisone Acetate and switched to Methylprednisolone for MN. Her respiratory status gradually improved, leading to extubation to high-flow oxygen on day 22. Furthermore, follow-up CT demonstrated significant resolution of pulmonary opacities (Figure 2b).

**FIGURE 3:**
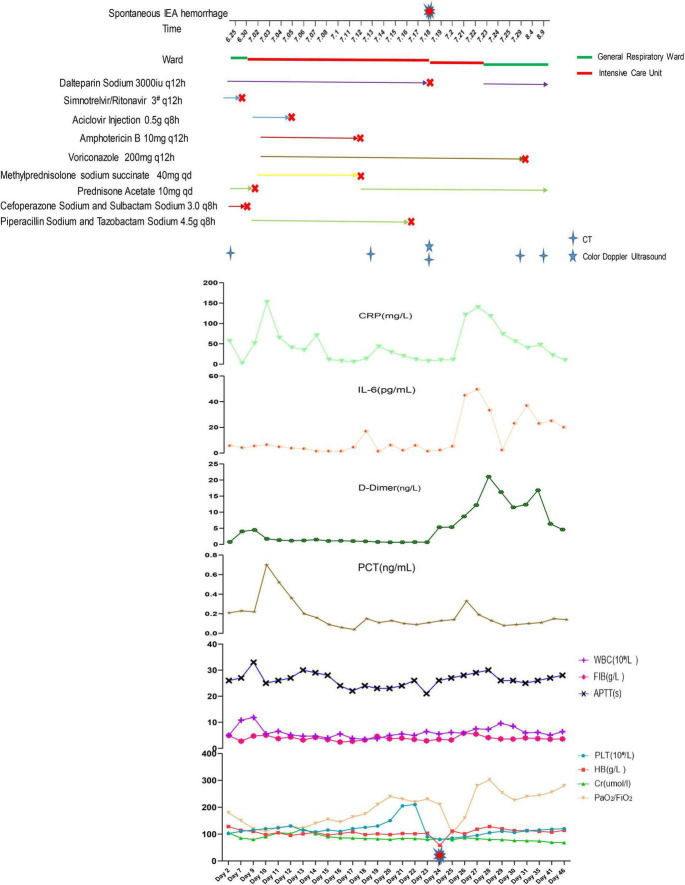
Patient’s clinical course. IEA, inferior epigastric artery; CRP, C-reactive protein; PCT, procalcitonin; WBC, white blood cell; FIB, fibrinogen; APTT, activated partial thromboplastin time; PLT, platelet; HB, hemoglobin; Cr, creatinine.

On day 24, she developed acute abdominal pain with a palpable right lower quadrant mass. Her hemoglobin dropped from 103 to 58 g/L, accompanied by hemorrhagic shock with a blood pressure of 70/40 mmHg and a heart rate of 125 beats per minute. Anticoagulation was discontinued. Bedside ultrasound (Figure 2c) and abdominal CT (Figure 2d) revealed a large rectus sheath hematoma with active extravasation. Emergency surgical exploration confirmed active bleeding from the right inferior epigastric artery, which was successfully ligated. Postoperatively, she required transient mechanical ventilation and transfusion support. Clinical stabilization permitted her transfer to the general ward on day 30. Subsequent chest CT imaging on day 35 demonstrated a significant resolution of pulmonary opacities (Figure 2e). At discharge on day 46, her respiratory symptoms had resolved, and follow-up chest CT showed significant improvement (Figure 2f). Three-months outpatient follow-up confirmed complete hematoma resolution, stable renal function and no recurrent bleeding or thrombotic events after receiving low-dose prednisolone. Anticoagulation was not resumed due to the prior major bleeding and resolved COVID-19. Patient’s clinical course was shown in Figure 3.

## Discussion

The management of COVID-19 is frequently complicated by coagulopathies that present a dual challenge: a pronounced risk of thrombosis alongside a non-negligible potential for hemorrhage (4, 5). This case illustrates a severe hemorrhagic complication–spontaneous inferior epigastric artery (IEA) rupture leading to a rectus sheath hematoma (RSH)–in a patient with COVID-19-associated ARDS and membranous nephropathy (MN) undergoing anticoagulant therapy. It underscores the critical need for meticulously balancing thromboprophylaxis against hemorrhage risk, particularly in patients with renal comorbidities.

Severe SARS-CoV-2 infection is associated with a profound inflammatory response that can trigger endothelial injury, hypercoagulability, and impaired fibrinolysis (9). The virus binds to Angiotensin-Converting Enzyme 2 receptors widely expressed on vascular endothelial cells, leading to endothelial dysfunction and a prothrombotic state. While venous thromboembolism (VTE) and microthrombosis are well-documented complications, emerging evidence indicates that bleeding events also occur with significant frequency, particularly in critically ill patients. A key pathophysiological mechanism involves dysregulation of the fibrinolytic system; elevated levels of both plasminogen activator inhibitor-1 (PAI-1) and tissue plasminogen activator (tPA) have been observed. This imbalance may predispose patients to simultaneous thrombotic and hemorrhagic events, especially when therapeutic anticoagulation is introduced (10, 11). Recent studies have reported an increase in spontaneous bleeding episodes among anticoagulated COVID-19 patients, even in some cases where anticoagulation was deemed “subtherapeutic” by conventional measures (7, 8, 12, 13). This suggests that COVID-19 itself may induce a unique coagulopathy where standard anticoagulation protocols could exacerbate bleeding risks (14–16). Our patient, despite having multiple risk factors for thrombosis (COVID-19 pneumonia, ARDS, and MN), experienced a major hemorrhagic event. This highlights that bleeding risk is not solely determined by anticoagulant dose but also by the underlying COVID-19-induced coagulopathy and patient-specific factors (7, 8). Notably, in patients with pre-existing renal conditions such as MN, this coagulopathic imbalance is further amplified due to the pre-existing vulnerability of the kidney. The kidney itself is a known target for SARS-CoV-2, which can directly infect renal cells expressing Angiotensin-Converting Enzyme 2 (ACE2) receptors, potentially exacerbating underlying renal pathology and contributing to a more complex hemodynamic and metabolic milieu (17).

The presence of MN significantly complicates anticoagulant therapy by altering its pharmacodynamics and pharmacokinetics. MN is itself a prothrombotic state due to urinary loss of anticoagulant proteins (e.g., antithrombin, proteins C and S) and often necessitates anticoagulation. However, renal impairment secondary to MN alters the pharmacokinetics of low-molecular-weight heparin (LMWH), which is primarily renally excreted (18, 19). This renal dysfunction can lead to reduced clearance and unexpected accumulation of LMWH, even at prophylactic doses, thereby effectively. For patients with a creatinine clearance <30 mL/min, clinicians usually consider using unfractionulated heparin (UFH) or a dose-adjusted LMWH regimen due to the increased risks of accumulation and bleeding. This patient received dalteparin 3000 IU q12 h, which might have led to drug accumulation and effectively created a “therapeutic” anticoagulant effect given her renal impairment. This argues strongly for the use of anti-Xa level monitoring to guide LMWH dosing in patients with renal dysfunction, a practice that is recommended but not always routinely implemented (15, 16). Furthermore, MN patients often require immunosuppressive regimens (e.g., corticosteroids, rituximab), which can further exacerbate bleeding risk (20, 21). Corticosteroids, while essential for controlling MN, contribute to vascular fragility by inhibiting fibroblast activity and collagen synthesis, impairing vascular integrity and wound healing (22). The patient’s switch from prednisone to methylprednisolone during her ICU stay might have further intensified this effect. Additionally, the chronic inflammatory state in MN can dysregulate endothelial function and trigger coagulation dysfunction (23).

The detection of *Aspergillus fumigatus* and HSV via tNGS in this critically ill, immunocompromised patient with COVID-19 highlighted a complex polymicrobial infection, necessitating an integrated antimicrobial strategy. Severe SARS-CoV-2 infection induces a state of immune dysregulation, and the administration of corticosteroids for managing comorbidities such as MN and COVID-19-related inflammation may further suppress host defenses, thereby fostering an opportunistic environment for diverse pathogens (24, 25). COVID-19-associated pulmonary aspergillosis (CAPA) is a well-documented and serious complication in critically ill patients, particularly those receiving immunosuppressive therapy or requiring mechanical ventilation, and is associated with increased mortality (26). Similarly, viral reactivations, including HSV, are frequent in severely ill or immunocompromised hosts and can contribute to pulmonary pathology, complicating the clinical course (27, 28). Distinguishing between colonization and invasive infection in this context requires careful clinical interpretation, a challenge extensively documented in CAPA literature (29). In the present case, the positive tNGS result for *Aspergillus fumigatus* was interpreted as indicative of probable infection rather than mere colonization. This assessment was based on the concurrent clinical context of progressive respiratory deterioration requiring intensive care, significant immunocompromise due to both MN therapy and severe COVID-19, and the initiation of targeted antifungal therapy. The decision to initiate voriconazole was consistent with guidelines endorsing it as a first-line agent for invasive aspergillosis/CAPA in critically ill patients (30, 31). Although mycological culture remains the gold standard for definitive diagnosis and susceptibility profiling, its results require several days (32). Given the patient’s rapid deterioration and the high mortality associated with untreated fungal infections in immunocompromised hosts, empirical voriconazole was initiated promptly upon tNGS detection, aligning with recommendations for pre-emptive therapy in high-risk patients with suggestive clinical and radiological features. While tNGS offers a rapid and sensitive tool for pathogen detection, its results must be integrated with radiological findings and host factors for accurate diagnosis. The simultaneous administration of broad-spectrum antibiotics addressed possible bacterial co-infection, following institutional protocols for hospital-acquired pneumonia in ventilated patients (33, 34). The subsequent clinical improvement following targeted antifungal and antiviral therapy underscores the appropriateness of the initial antimicrobial selection and highlights the importance of early, broad-spectrum coverage in critically ill immunocompromised patients, with subsequent de-escalation based on microbiological results and clinical response. This case emphasizes the critical role of proactive microbiological surveillance, including bronchoscopic evaluation when indicated, and underscores the necessity of a multidisciplinary approach involving intensivists, microbiologists, and infectious disease specialists to interpret complex tNGS findings and guide appropriate antimicrobial therapy in critically ill ICU patients.

The spontaneous nature of the IEA hemorrhage in this patient is a critical point that can be understood within this framework of COVID-19-associated endothelial injury, coagulopathy, and MN-altered drug pharmacokinetics. It is highly unlikely that this was a direct consequence of LMWH subcutaneous injection. Anatomically, the IEA runs deep within the posterior rectus sheath. Subcutaneous injections of LMWH are administered into the adipose tissue anterior to the rectus sheath, using short needles (typically 4–6 mm for prefilled syringes) designed specifically to avoid intramuscular injection and vascular injury (35). The IEA, being a deeper structure, is not a plausible target for a standard injection. Therefore, the hemorrhage is far more likely attributable to the anticoagulated state itself, potentially compounded by COVID-19-associated endothelial dysfunction and the vascular fragility that can accompany chronic steroid use or the inflammatory state of MN (8, 11–13, 16, 36, 37). A sudden increase in intra-abdominal pressure from coughing or straining–common in COVID-19 pneumonia–could have been the precipitating event in the setting of altered coagulation.

This case underscores several key lessons for managing complex COVID-19 patients. First, embrace individualized anticoagulation: a one-size-fits-all approach is inadequate. Anticoagulation must be tailored based on thrombosis risk, bleeding risk scores, and renal function. Second, mandate pharmacodynamic monitoring: in patients with renal impairment, monitoring anti-Xa levels for LMWH activity is crucial to avoid inadvertent over-anticoagulation. Third, maintain high clinical suspicion for bleeding: in anticoagulated COVID-19 patients presenting with acute abdominal pain or anemia, RSH must be considered in the differential diagnosis. Point-of-care ultrasound can be a rapid and effective tool for initial screening. Fourth, adopt a multidisciplinary approach: decision-making for such patients should involve intensivists, nephrologists, and hematologists to carefully weigh the competing risks of thrombosis and hemorrhage.

While this case provides valuable insights into the management of anticoagulation in a complex COVID-19 patient with membranous nephropathy, several limitations should be acknowledged. First, as a single-case report, our findings describe the experience of one individual at a single center. The observations and management strategies reported here, while informative, may not be directly generalizable to all patients with similar clinical profiles. Second, the retrospective nature of the data collection, inherent to most case reports, means that some laboratory monitoring, such as serial anti-Xa levels to precisely track dalteparin activity, was not performed. The absence of this pharmacokinetic data limits our ability to make definitive conclusions about the exact degree of anticoagulation accumulation. Furthermore, the patient’s complex clinical course, involving sequential interventions for COVID-19 and superimposed infections, introduces multiple potential confounding factors that may have influenced the outcome. Finally, the medium-term follow-up of 3 months, while showing no recurrent events, may not capture the long-term risks of thrombosis after anticoagulation withdrawal. Future prospective studies with larger cohorts are warranted to validate the associations suggested by this case and to develop more precise risk-stratification tools for this high-risk population.

## Conclusion

The management of COVID-19 in patients with pre-existing renal disease like MN requires extremely careful consideration. While anticoagulation remains a cornerstone of management to prevent thrombotic complications, this case serves as a critical reminder of its potential to cause severe hemorrhage. Future studies should focus on developing validated algorithms for personalized anticoagulation in this high-risk population to optimally navigate the fine line between thromboprophylaxis and bleeding risk.

## Data Availability

The original contributions presented in this study are included in this article/supplementary material, further inquiries can be directed to the corresponding author.
